# Supplementary material for: Trends of proximate low birth weight and associations among children under-five years of age: Evidence from the 2016 Ethiopian demographic and health survey data
Source: PLoS One. 2021 Feb 10;16(2):e0246587. doi: 10.1371/journal.pone.0246587 (PMC7875362; doi:10.1371/journal.pone.0246587)
Supplement: Attachment — Submitted filename: Review_31-10-20.docx [file pone.0246587.s001.docx]

I really appreciate the efforts by the author(s) on this manuscript but I feel the paper is not yet ready for publication in current shape and needs some major revisions. My comments are as follows:

1. The introduction should aim to contextualize the topic for the empirical analysis from the evidence with focus on similar studies from Ethiopia that were conducted in recent years.
2. The authors should present bivariate analysis to see the relationship and outcome of independent and dependent variables rather than directly attempting the regression analysis. This analysis will help understand the correlations across determinants and its association with low birth weight.
3. I also found the author(s) is missing many of the maternal health factors which are affecting the birth weight. For example, maternal BMI, anemia status, maternal nutrition and indicators such as antenatal care etc.
4. The policy implications of the findings should be discussed in the context of the literature and policy focus in Ethiopia.
5. The trend analysis should be highlighted in the introduction or in the discussion section.

Author should incorporate the comments and revise the manuscript and submit again as I think the topic has potential to be published but not in current form.
