# Supplementary material for: Trends of proximate low birth weight and associations among children under-five years of age: Evidence from the 2016 Ethiopian demographic and health survey data
Source: PLoS One. 2021 Feb 10;16(2):e0246587. doi: 10.1371/journal.pone.0246587 (PMC7875362; doi:10.1371/journal.pone.0246587)
Supplement: Attachment — Submitted filename: Point by point feedback-to reviewers.docx [file pone.0246587.s002.docx]

**Responses for reviewer comments**

First of all, we would like to say thank you for your constructive and very important comments.

**General comments**

We accept the comments and take appropriate changes. We have also edited the language

**Reviewer #1:**

1. In abstract, result is not presented properly instead only statistics are given. Please write the major findings in sentence.

We write the prevalence, and the variation in child size. In addition we have also listed the associated variables. But we may not able to show the findings clearly. The major findings are presented with major statistics.

2. In method section you have listed a set of characteristics but you have not given any justification why you refer to these characteristics, are they from your literature study, if so mention clearly. Please highlight the specific research gap you have tried to address in your work.

We select the characteristics based on literature but if the variables we select are not available at the EDHS data, we forced to omit it. We tried to add the gaps regarding this research in last paragraph of the introduction part.

3. The result section is presented in a casual manner and table statistics were presented only. Please write in words what they imply.

We write some of the contents of the table in narration as per the comment. But we are unable to write all the contents from the table to text.

4. If information on birth weight is available in the data set then can you use this variable as birth size may be subjective as it is based on perception of the mother may be or that of respondent.

The birth weight in the data set is not complete. There are many missing variables even mothers cannot remember the exact birth weight but they can remember the size easily. And almost many of mothers gave a date regarding the average size. But, if there is a data of birth weight, we did not use it because our operational definition does not consider it. We take the data reported from mothers or care givers as per the EDHS inclusion criteria

5. The set of explaining variables in the table 2 does not give justification why you choose them, instead you can run a factor analysis and find out the most important ones. Some of these explaining variables might have association between them.

Sorry for missing the clarification of variable selection to multivariate analysis but we included in this version at the first paragraph, first line of factors associated with proximate LBW section. We select variables that have a p-value of 0.25 or below, and we run mlticollinearity test between close variables based on literature and authors knowledge. In this scenario, Khat chewing and cigarette smoking were had collinearity. Thus, we select Khat chewing to the multivariate analysis. However, none of the other variables have no collinearity and we tried some of the variables randomly.

6. I suggest authors should use factor analysis to identify which explaining variables are related to the individual characteristics (maternal education, khat chewing, child sex, sex of the child, child birth type etc), household characteristic (wealth, residence, religion etc) and policy variable (place of delivery, delivery attended by whom?).

We accept the comment and we tried a multicollinearity test for the association in between them. In the entire variables, there was no association as the Tolerance (VIF) indicates except Khat chewing and cigarette smoking.

7. A justification for using binomial logit can be provided.

The outcome variables are binary, and fulfill the assumption of logistic regression like normality test, and large sample size, and model fitness test. We included this justification in the data analysis section.

8. The language in the text needs to be refined. Please take help of professional to refine the text. For example what do you mean by 'child sex', instead sex of the child would be better.

Accepted and get an English language professional for final edition.

9. In conclusion section there is no reference to how this research contributes to policy for child health. Please include some policy implication.

Accepted and try to include a policy implication using data from Minister of Health, Ethiopia and other find. We have also cited as per the comment.

10. Your region variable is very important and you have not shown how different regions in Ethiopia vary in socioeconomic profile. It will help to build context for regional variation in your analysis

Thank you, most of the regions have association with PLBW as our multivariate analysis indicates but we did not describe it as per the socio-economic perspective. However, we include a justification with citation in the discussion part.

11. Please give the model statistics to show the robustness of your model fit.

We include it in data analysis section that the model is fitted as per the Hosmer-lemeshow-goodness of fit test.

12. Please write a section on policies adopted in Ethiopia on child health and what can be done to improve them for better child health outcome in terms of birth weight and size of the child at birth.

We have added such section in the background

**Reviewer #2:**

1. The introduction should aim to contextualize the topic for the empirical analysis from the evidence with focus on similar studies from Ethiopia that were conducted in recent years.

We accept the comment and we cited some studies that conducted in Ethiopia.

2. The authors should present bivariate analysis to see the relationship and outcome of independent and dependent variables rather than directly attempting the regression analysis. This analysis will help understand the correlations across determinants and its association with low birth weight.

In table two, we include the cross-tab result, the binary result (COR), and the multilevel regression (AOR) result. We did not directly run multilevel analysis, but to include the details of the binary result, the table cannot be managed. However, we include the most important components even for authors who want to done meta-analysis we include the cross-tab (A, B, C & D) variables. For selecting variables for multivariate analysis based on COR, we indicate in (*). Thus, we cannot manage all the binary and multivariate results in one table rather we prefer to present the most important contents based on some other researches.

3. I also found the author(s) is missing many of the maternal health factors which are affecting the birth weight. For example, maternal BMI, anemia status, maternal nutrition and indicators such as antenatal care etc.

I really sorry for not answering this question by adding the variables, but these variables were excluded at the beginning of the study when we refine the data. The major limitation of DHS analysis is losing such important variables. When we select the outcome variables, we forced to lose some variables simultaneously. In addition, some of variables like BMI excluded because of missing data on the variable.

4. The policy implications of the findings should be discussed in the context of the literature and policy focus in Ethiopia.

We accepted it and include a policy indication in the conclusion section with citation

5. The trend analysis should be highlighted in the introduction or in the discussion section.

We tried to indicate the trend as follow

The aim of this study was to assess the trends of proximate LBW and associations from 2011 to 2016 in Ethiopia using the 2016 EDHS data. Since the survey had 5 years compressive data, it can indicate the trends better than the other primary and area specific studies.
