# Supplementary material for: Trends of proximate low birth weight and associations among children under-five years of age: Evidence from the 2016 Ethiopian demographic and health survey data
Source: PLoS One. 2021 Feb 10;16(2):e0246587. doi: 10.1371/journal.pone.0246587 (PMC7875362; doi:10.1371/journal.pone.0246587)
Supplement: Attachment — Submitted filename: Responses for reviewer comments.docx [file pone.0246587.s003.docx]

**Responses for reviewer comments**

First of all, we would like to say thank you for your constructive and very important comments.

**I included the following sentences as per the reviewer comments**

**You can give the equation for your logistic model in the final version of the paper.**

1.

**In the introduction write few sentences about how this research is bridging any existing gap in the available research on the topic.**

**2.** Because of that most previous conducted studies are limited geographically, by population, and sample size. In addition, those studies are single shot and did not show the trends of low birth weight which might be susceptible to bias because of drought or other seasonal factors like political instability and natural disaster. However, this study considered a 5 consecutive year’s data to assess the trends and potential determinants of proximate low birth weight.

**Also write bit more about policy implication of your paper**

3. Thus, this paper will be used by ministry, health facilities, and volunteer organizations to develop and amend policies to treat or prevent low birth weight in the country.

Thank you!!
